# Supplementary material for: Perception and coping mechanisms of patients with diabetes mellitus during the COVID-19 pandemic in Ibadan, Nigeria
Source: PLoS One. 2024 Aug 27;19(8):e0309451. doi: 10.1371/journal.pone.0309451 (PMC11349176; doi:10.1371/journal.pone.0309451)
Supplement: Attachment — Submitted filename: PONE-D-23-18592_Reviewer comments.doc [file pone.0309451.s003.doc]

**PLOS ONE**

**Perception and coping mechanisms among diabetes mellitus patients at the University College Hospital, Ibadan, Nigeria, during the COVID-19 pandemic**

--Manuscript Draft--

| **Manuscript Number:** | PONE-D-23-18592 |
| --- | --- |
| **Article Type:** | Research Article |
|  |  |
| **Full Title:** | Perception and coping mechanisms among diabetes mellitus patients at the University |
|  | College Hospital, Ibadan, Nigeria, during the COVID-19 pandemic Consider rewording the title to: Perceptions and coping mechanisms of patients with diabetes mellitus during COVID 19 pandemic in Ibadan Nigeria |
| **Short Title:** | Perception and coping mechanisms among diabetes mellitus patients during the |
|  | COVID-19 pandemic in Nigeria |
|  |  |
| **Corresponding Author:** | Obasanjo Afolabi Bolarinwa, Masters |
|  | University of KwaZulu-Natal College of Health Sciences |
|  | Durban, KwaZulu-Natal SOUTH AFRICA |
| **Keywords:** | Covid-19; pandemic; Diabetes mellitus; Vulnerable; Nigeria |
|  |  |
| **Abstract:** | Background |
|  | The 2019 coronavirus disease (COVID-19) pandemic ushered in a period of fear and |
|  | uncertainty, resulting in structural instability across the globe. Vulnerable individuals, |
|  | such as patients with diabetes mellitus (DM), are bound to have adverse effects and |
|  | complications of COVID-19 when infected. We sought to understand the perception of |
|  | DM patients during the COVID-19 pandemic and their coping mechanisms at the |
|  | University College Hospital, Ibadan. |
|  | Methods |
|  | We employed an exploratory qualitative study design to understand DM patients’ |
|  | perceptions and coping mechanisms with COVID-19. A purposive sampling technique |
|  | was used to recruit 32 participants (2 health professionals and 30 DM patients). In- |
|  | depth interviews were used to collect the data from the participants. All the audio data |
|  | were transcribed verbatim and exported to NVivo software for data analyses. |
|  | Results |
|  | Most DM patients were not fearful of the pandemic but were optimistic that it would not |
|  | affect their health. Mechanisms such as the usage of herbal medicines and adherence |
|  | to COVID-19 precautionary measures were noticed among patients. The study also |
|  | revealed the hospital's coping mechanism during the COVID-19 pandemic: prolonged |
|  | appointments, limiting the number of patients attended per clinic day, and the provision |
|  | of telehealth service. Patients in our study utilized negative coping mechanisms such |
|  | as reduced drug dosages, subscriptions to cheaper drug brands, and reliance on |
|  | religious institutions rather than a clinic for health instructions. |
|  | Conclusions |
|  | The study has shown that DM patients were not fearful of the COVID-19 pandemic |
|  | ~~despite their condition~~. The utilisation of telehealth, encouragement of daily monitoring |
|  | of sugar levels, provision of avenues for a medication review, and adherence to the |
|  | safety protocols were coping mechanisms employed by the health system and the DM |
|  | patients. We recommend that the government and other healthcare stakeholders must reinforce the |
|  | resilience of DM patients by alleviating their health burdens during the pandemic. This |
|  | could be done by subsidizing the prices of drugs, tests, and consultation fees for the patients with DM., |
|  | Improving the waiting and appointment system in the clinic , creating an online |
|  | presence for the UCH Diabetes Association Office, and employing more health |
|  | personnel in the clinic. |
| **Order of Authors:** | Olajumoke Ololade Tunji-Adepoju, Masters |
|  |  |
|  | Obasanjo Afolabi Bolarinwa, Masters |
|  |  |
|  |  |

Powered by Editorial Manager® and ProduXion Manager® from Aries Systems Corporation

|  | Richard Gyan Aboagye, Masters |
| --- | --- |
|  |  |
|  | Williams O Balogun |
|  |  |
| **Additional Information:** |  |
| **Question** | **Response** |
|  |  |
| **Financial Disclosure** | The author(s) received no specific funding for this work. |

Enter a financial disclosure statement that

describes the sources of funding for the

work included in this submission. Review

the [submission guidelines](http://journals.plos.org/plosone/s/submission-guidelines#loc-financial-disclosure-statement) for detailed

requirements. View published research

articles from [*PLOS ONE*](http://journals.plos.org/plosone/) for specific

examples.

This statement is required for submission

and **will appear in the published article** if

the submission is accepted. Please make

sure it is accurate.

**Unfunded studies**

Enter: *The author(s) received no specific*

*funding for this work.*

**Funded studies**

Enter a statement with the following details:

- Initials of the authors who received each award
- Grant numbers awarded to each author
- The full name of each funder
- URL of each funder website
- Did the sponsors or funders play any role in the study design, data collection and analysis, decision to publish, or preparation of the manuscript?
- **NO** - Include this sentence at the end of your statement: *The funders had no role in study design, data collection and analysis, decision to publish, or preparation of the manuscript.*
- **YES** - Specify the role(s) played.
- typeset

| **Competing Interests** | The authors have declared that no competing interests exist. |
| --- | --- |

Use the instructions below to enter a

competing interest statement for this

submission. On behalf of all authors,

disclose any [competing interests](http://journals.plos.org/plosone/s/competing-interests) that

could be perceived to bias this

work—acknowledging all financial support

and any other relevant financial or non-

*Powered by Editorial Manager® and ProduXion Manager® from Aries Systems Corporation*

financial competing interests.

This statement is required for submission

and **will appear in the published article** if

the submission is accepted. Please make

sure it is accurate and that any funding

sources listed in your Funding Information

later in the submission form are also

declared in your Financial Disclosure

statement.

View published research articles from

[*PLOS ONE*](http://journals.plos.org/plosone/) for specific examples.

**NO authors have competing interests**

Enter: *The authors have declared that no*

*competing interests exist.*

**Authors with competing interests**

Enter competing interest details beginning

with this statement:

*I have read the journal's policy and the*

*authors of this manuscript have the following*

*competing interests: [insert competing*

*interests here]*

* typeset

| **Ethics Statement** | N/A |
| --- | --- |

Enter an ethics statement for this

submission. This statement is required if

the study involved:

- Human participants
- Human specimens or tissue
- Vertebrate animals or cephalopods
- Vertebrate embryos or tissues
- Field research

Write "N/A" if the submission does not

require an ethics statement.

General guidance is provided below.

Consult the [submission guidelines](http://journals.plos.org/plosone/s/submission-guidelines#loc-guidelines-for-specific-study-types) for

*Powered by Editorial Manager® and ProduXion Manager® from Aries Systems Corporation*

detailed instructions. **Make sure that all**

**information entered here is included in the**

**Methods section of the manuscript.**

**Format for specific study types**

**Human Subject Research (involving human**

**participants and/or tissue)**

- Give the name of the institutional review board or ethics committee that approved the study
- Include the approval number and/or a statement indicating approval of this research
- Indicate the form of consent obtained (written/oral) or the reason that consent was not obtained (e.g. the data were analyzed anonymously)

**Animal Research (involving vertebrate**

**animals, embryos or tissues)**

- Provide the name of the Institutional Animal Care and Use Committee (IACUC) or other relevant ethics board that reviewed the study protocol, and indicate whether they approved this research or granted a formal waiver of ethical approval
- Include an approval number if one was obtained
- If the study involved *non-human primates*, add *additional details* about animal welfare and steps taken to ameliorate suffering
- If anesthesia, euthanasia, or any kind of animal sacrifice is part of the study, include briefly which substances and/or methods were applied

**Field Research**

Include the following details if this study

involves the collection of plant, animal, or

other materials from a natural setting:

- Field permit number
- Name of the institution or relevant body that granted permission

| **Data Availability** | Yes - all data are fully available without restriction |
| --- | --- |

Authors are required to make all data

underlying the findings described fully

available, without restriction, and from the

time of publication. PLOS allows rare

Powered by Editorial Manager® and ProduXion Manager® from Aries Systems Corporation

| exceptions to address legal and ethical | | | | |  |
| --- | --- | --- | --- | --- | --- |
| concerns. See the [PLOS Data Policy](http://journals.plos.org/plosone/s/data-availability) and | | | | |  |
|  | |  |  |  |  |
| [FAQ](http://journals.plos.org/plosone/s/data-availability#loc-faqs-for-data-policy) for detailed information. | | | | |  |
|  | |  | | |  |
| A Data Availability Statement describing | | | | |  |
| where the data can be found is required at | | | | |  |
| submission. Your answers to this question | | | | |  |
| constitute the Data Availability Statement | | | | |  |
| and **will be published in the article**, if | | | | |  |
| accepted. | | | | |  |
|  | |  |  |  |  |
| **Important:** Stating ‘data available on request | | | | |  |
| from the author’ is not sufficient. If your data | | | | |  |
| are only available upon request, select ‘No’ for | | | | |  |
| the first question and explain your exceptional | | | | |  |
| situation in the text box. | | | | |  |
| Do the authors confirm that all data | | | | |  |
| underlying the findings described in their | | | | |  |
| manuscript are fully available without | | | | |  |
| restriction? | | | | |  |
| **Describe where the data may be found in** | | | | | The data underlying the results presented in the study are available upon request |
| **full sentences. If you are copying our** | | | | | through the corresponding author. |
| **sample text, replace any instances of XXX** | | | | |  |
| **with the appropriate details.** | | | | |  |
| • | If the data are **held or will be held in a** | | | |  |
|  | **public repository**, include URLs, | | | |  |
|  | accession numbers or DOIs. If this | | | |  |
|  | information will only be available after | | | |  |
|  | acceptance, indicate this by ticking the | | | |  |
|  | box below. For example: *All XXX files* | | | |  |
|  | *are available from the XXX database* | | | |  |
|  | *(accession number(s) XXX, XXX.*). | | | |  |
| • | If the data are all contained **within the** | | | |  |
|  | **manuscript and/or Supporting** | | | |  |
|  | **Information files**, enter the following: | | | |  |
|  | *All relevant data are within the* | | | |  |
|  | *manuscript and its Supporting* | | | |  |
|  | *Information files.* | | | |  |
| • If neither of these applies but you are | | | | |  |
|  | able to provide **details of access** | | | |  |
|  | **elsewhere**, with or without limitations, | | | |  |
|  | please do so. For example: | | | |  |
|  | *Data cannot be shared publicly because* | | | |  |
|  | *of [XXX]. Data are available from the* | | | |  |
|  | *XXX Institutional Data Access / Ethics* | | | |  |
|  | *Committee (contact via XXX) for* | | | |  |
|  | *researchers who meet the criteria for* | | | |  |
|  |  |  |  |  |  |

*Powered by Editorial Manager® and ProduXion Manager® from Aries Systems Corporation*

*access to confidential data.*

*The data underlying the results*

*presented in the study are available*

*from (include the name of the third party*

*and contact information or URL).*

- This text is appropriate if the data are owned by a third party and authors do not have permission to share the data.

* typeset

Additional data availability information:

*Powered by Editorial Manager® and ProduXion Manager® from Aries Systems Corporation*

Manuscript

[Click here to](https://www.editorialmanager.com/pone/download.aspx?id=33571264&guid=d4ff0edf-8fee-4ef7-b8fd-9a62b8b9a31e&scheme=1)

[access/download;Manuscript;Manuscript_Diabetes_Managemen](https://www.editorialmanager.com/pone/download.aspx?id=33571264&guid=d4ff0edf-8fee-4ef7-b8fd-9a62b8b9a31e&scheme=1)

1 **”: Perception and coping**
2 **mechanisms among diabetes mellitus patients at the University College Hospital, Ibadan,**

3	**Nigeria, during the COVID-19 pandemic.**

4 Olajumoke Ololade Tunji-Adepoju^1^, Obasanjo Afolabi Bolarinwa ^2,3*^, Richard Gyan Aboagye^3^,
5 Williams O Balogun ^4^
6 ^1^Department of Sociology, University of Ibadan, Ibadan, Nigeria.
7 ^2^Department of Public Health Medicine, School of Nursing and Public Health, University of
8 KwaZulu-Natal, Durban, South Africa.
9 ^3^Department of Public Health, York St John University, London, United Kingdom.
10 ^4^Department of Family and Community Health, Fred N. Binka School of Public Health,
11 University of Health and Allied Sciences, Hohoe, Ghana.
12 ^5^Endocrinology and Diabetes Division, Department of Internal Medicine, College of Medicine,
13 University of Ibadan & University College Hospital, Ibadan, Nigeria.

14

15 ***Correspondence**
16 tunadejum@gmail.com
17 wobalogun@yahoo.com
18 raboagye18@sph.uhas.edu.gh
19 *bolarinwaobasanjo@gmail.com

20

1

21 **Abstract**
22 **Background**
23 The 2019 coronavirus (COVID-19) pandemic ushered in a period of fear and
24 uncertainty, resulting in structural instability across the globe. Vulnerable individuals, such as
25 patients with diabetes mellitus (DM), are bound to have adverse effects and complications of
26 COVID-19 when infected. We sought to understand the perception of DM patients during the
27 COVID-19 pandemic and their coping mechanisms at the University College Hospital, Ibadan.
28 **Methods**
29 We employed an exploratory qualitative study design to understand DM patients’ perceptions
30 and coping mechanisms with COVID-19. A purposive sampling technique was used to recruit
31 32 participants (2 health professionals and 30 DM patients). In-depth interviews were used to
32 collect the data from the participants. All the audio data were transcribed verbatim and
33 exported to NVivo software for data analyses.
34 **Results**
35 Most DM patients were not fearful of the pandemic but were optimistic that it would not
36 affect their health. Mechanisms such as using herbal medicines and adherence to COVID-19
37 precautionary measures were noticed among patients. The study also revealed the hospital's
38 coping mechanism during the COVID-19 pandemic: prolonged appointments, limiting the
39 number of patients attended per clinic day, and providing telehealth service. Patients in our
40 study utilized negative coping mechanisms such as reduced drug dosages, subscriptions to
41 cheaper drug brands, and reliance on religious institutions rather than a clinic for health
42 instructions.
43 **Conclusions**
44 The study concluded that despite their condition, DM patients were not fearful of the COVID-
45 19 pandemic. The utilisation of telehealth, encouragement of daily monitoring of sugar levels,
46 provision of avenues for a medication review, and adherence to the safety protocols were
47 coping mechanisms employed by the health system and the DM patients. The government

2

48 and other healthcare stakeholders must reinforce the resilience of DM patients by alleviating
49 their health burdens during the pandemic. This could be done by subsidizing the prices of
50 drugs, tests, and consultation fees, improving the waiting and appointment system in the
51 clinic, creating an online presence for the UCH Diabetes Association Office, and employing
52 more health personnel in the clinic.

53

54 **Keywords:** COVID-19, Pandemic, Diabetes Mellitus, Patients Vulnerable, Nigeria

55

56

57

58

59

60

61

62

63

64

65

66

67

68

69

3

70 **Background**
71 Diabetes Mellitus (DM) has been an important global health concern even prior to the
72 outbreak of the 2019 coronavirus disease (COVID-19) pandemic. DM is a chronic health
73 condition characterized by high blood glucose due to either inability of the pancreas to
74 produce adequate insulin or the body's resistance to available insulin [1]. Poorly managed DM
75 can result in long-term complications such as amputation, cardiovascular disease, vision
76 impairment, and renal disease [1]. Sadly, the prevalence of DM is increasing across the globe.
77 Between 1980 and 2014, the number of patients with DM increased from 108 million to 422 million [2] and over 500
78 million in 2020 [3, 4]. DM is projected to be the seventh leading cause of death worldwide by
79 2030 [2]. The burden of diabetes is greatly increasing in sub-Saharan Africa, and Nigeria has
80 the largest share of this burden [5, 6]. Studies estimated that about 11 million Nigerians have
81 diabetes [7]. This implies that more than five percent of the Nigerian population is affected by
82 the disease, and it is a major cause of death among many Nigerians below the age of 70 [8].
83 Research has shown that the presence of excess blood glucose, also known as hyperglycemia

nclu

84 in diabetic patients, makes them susceptible to COVID-19 include reference. In the same vein, studies ihave also Please check the use of “diabetis patients” and change toeither patients with diabetis OR diabetic patients”. Check the entire document and correct
85 shown that patients with diabetes are two times more likely to develop severe conditions or
86 die from COVID-19, while people with uncontrolled diabetes are about 13 times more likely to
87 die from the virus [9]. This has instilled fear in many diabetes patients in Nigeria [10]. Hence,
88 the fact that diabetic patients need extra health care and attention during the pandemic
89 remains undisputable [11]. Measures to prevent and control Non-Communicable Diseases
90 (NCDs), such as diabetes, in developing countries, including Nigeria, have not been effectively
91 implemented. The COVID-19 pandemic worsened the accumulated effects of Nigeria’s failure
92 to improve the healthcare structure and system, particularly the care of people with NCDs.
93 The outbreak of COVID-19 in Nigeria on the 27th of February 2020 [12] caused great
94 dysfunctionality and complications in the Nigerian social structures, requiring sporadic
95 responses.
96 Predictably, the response to COVID-19 in Nigeria infringed on treating other diseases,
97 including DM [13]. For instance, many public hospitals were converted to COVID-19 treatment

4

98 centers resulting in restricted access of other patients to medical care [5, 14]. The report of
99 the assessment of the continuity of essential health services during the pandemic carried out
100 by the World Health Organization (WHO) in its member states between 15th May 2020 and
101 29th July 2020 reveals the absence of equity in terms of the treatment dispensed in
102 eradicating COVID-19 and managing other diseases [4, 15]. More than fifty percent of its
103 member states nexperienced a disruption in delivering health care services on NCDs, cancer,
104 and mental health disorders. Some of the reasons given for this disruption included shortage of staff due
105 to their transfer to COVID-19 care centers, lack of public transport, and cancellation of planned
106 appointments, while the reasons recorded in 20% of the member states were: the shortage of
107 medicines, diagnostics, and other technologies [4].
108 Due to the various forms of hardship or discomfort caused by the pandemic, vulnerable
109 individuals such as people with DM are likely to perceive the period differently than healthy
110 people. Diabetic patients bare likely to have a negative perception of the COVID-19 condition,
111 which could influence the adoption of harmful coping mechanisms that could further hamper
112 their health outcomes. Although several perception studies have been done on COVID-19
113 generally in Nigeria, such as that of Osahon and Memudu [16] on the Perception and Healthy
114 Attitudes of Nigerians to COVID-19, very little data is available about the perception of
115 diabetes patients of the COVID-19 pandemic. This study aimed at capturing the perceptions
116 of DM patients during the pandemic and their coping mechanisms during the period. This is
117 important to illuminate the experiences of DM patients during the pandemic The findings
118 could be beneficial to the government and healthcare stakeholders in formulating policies to
119 help improve the health outcomes of diabetes patients during the pandemic.

120

121 **Methods**
122 **Study design**
123 We adopted an explorative qualitative research approach. This approach enabled the
124 researchers to understand the perception of DM patients at University College Hospital (UCH)

5

125 and the coping mechanisms they employed during the pandemic. This approach is right for
126 this study, as it helped to collect in-depth data from the participants in their natural form.

127

128 **Study setting**
129 The study was conducted in the UCH, Ibadan. UCH is a tertiary health center that serves Oyo
130 state and neighbouring states where patients with diabetes, including those with suspected
131 COVID-19 cases, are seen. According to statistics from the Nigeria Centre for Disease Control
132 and Prevention (NCDC) [17], Oyo State is among the COVID-19 most infected states in Nigeria,
133 and Ibadan is its capital city. The UCH was founded in November 1952, located at Oritamefa in
134 Ibadan North Local Government Area. It is the first teaching hospital in Nigeria to provide in-
135 patient and outpatient health care services. It receives referrals from southwestern and other
136 parts of Nigeria and outside the country. The Diabetes clinic runs every Monday, and an
137 average of 50-70 patients visit the clinic. One of the recommendations is employing more HCPs. Include the number of HCPs working at the outpatient clinics where patients with DM are seen.

138

139 **Participant sampling and sample size**
140 Participants in this study were health professionals (an endocrinologist and a nurse) and
141 registered diabetes patients who had been visiting the outpatient diabetes clinic in UCH
142 before the pandemic and are still attending the clinic during the pandemic. We used a
143 purposive samplingtechnique to recruit the participants for the studyi. These participants
144 constituted those who were knowledgeable and experienced the phenomena under study.
145 Only those who were living with DM before the pandemic were included for the patients.
146 However, only those accessible during the data collection period were included. The rationale
147 was that this category of DM patients could tell the difference between their experiences in
148 the clinic before and during the pandemic. Only individuals with a history of diagnosis of DM
149 at the UCH before the outbreak of the COVID-19 pandemic and who attended the diabetes
150 clinic during the period of the study were included in the study. Being coherent, healthy, gave
151 informed consent were other inclusion criteria for the study. Patients who did not consent to
152 the study or were no longer interested in participating even while in the middle of it were

6

153 excluded from the study. Patients diagnosed with diabetes after the outbreak of COVID-19
154 and/or who did not attend the diabetes clinic during the study period were excluded. Also,
155 the study did not include patients with acute or chronic debilitating comorbidities. Two health
156 professionals and 30 DM patients were included in the study.

157

158 **Data collection Please include an interview guide as a supporting document**
159 Four (4) trained research assistants were used for the data collection. The research assistants
160 were trained anthropologists, who the authors also trained in areas consisting of the
161 consenting process, interviews and asking probing questions, and recording. The authors
162 developed an interview guide as the data collection tool. Data on the participants'
163 sociodemographic characteristics and the study objectives were collected after obtaining consent. The
164 probe questions were structured to capture responses to the participants' perceptions of the
165 COVID-19 pandemic. It also covered responses to the coping mechanisms adopted by DM
166 patients during the pandemic. Prior to the data collection, we informed the participants about
167 any possible discomfort, benefits, and compensation associated with the study. Interviews
168 were conducted once the participants agreed to a date, time, and place of convenience to
169 participants. We conducted all of the interviews at the premises of the hospital. The data
170 collection lasted for an average of 60 minutes per participant. About 16 interviews were
171 conducted each week for two weeks by one of the researchers with four trained
172 anthropologists who had qualitative fieldwork experiences. Interviews were conducted with
173 strict adherence to the COVID-19 precautionary measures. The data collection/interviews
174 were done between the 15th to 22nd of March 2021. It is not clear how the participants were recruited. Include information on audio recording and field notes. Data saturation? Was there any pilot testing?

175

176

177 **Data analysis**
178 All the audio data was transcribed verbatim on the same day the data was collected. This
179 enabled the research assistants to identify questions that may have been left unanswered
180 during the interview or informed the research assistants of means to get detailed responses
181 per questions in subsequent interviews. After the transcription by the research assistants, the
182 transcripts and the recorded audio were carefully listened to and vetted by the authors. The
183 transcripts were later imported into NVivo software analysis. The analysis began by reading
184 all the imported transcripts for familiarization with the data. Afterwards, codes were assigned
185 to the text data. Recurring codes that were similar were categorised to generate themes and

7

186 sub-themes. We presented the extracts and quotes as the findings from the study. All the
187 authors approved all the extracts and quotes.

188

189 **Rigour and trustworthiness**
190 In every qualitative study, credibility and trustworthiness measures are critical. In achieving
191 this, we allowed two research assistants with experience in qualitative analysis to transcribe
192 and analyze tape-recorded interviews separately. The two research assistants' themes and
193 sub-themes and the authors were compared to ascertain their consistency. Direct quotations
194 and precise summaries of participant remarks were used to strengthen the credibility of the
195 results.
196 **Ethical issues**
197 Ethical clearance was sought from the Ibadan/University College Hospital Ethics Committee
198 (UI/UCH EC) with approval number: UI/EC/21/0064. In this study, we complied with all the
199 ethical guidelines pertaining to using human participants and peculiar to qualitative studies.
200 We anonymized all the transcripts and audio files by giving them pseudonyms to remove any
201 personal information that may be used to identify the study participants. The participants in
202 the study were given an information sheet that included information on the objectives,
203 methods, potential risks and advantages, compensations, who to contact, and an affirmation
204 of confidentiality, privacy, and autonomy. The participants gave written consent by signing
205 the informed consent form , and no participant under 18 years of age was involved in this
206 study. This demonstrated that they had read and understood the terms of reference before
207 participating freely in our study. We also encrypted a passcode and locked the audio files and
208 transcripts to prevent unauthorised individuals from accessing the material.

209

210 **Results**
211 Key informant interviews were conducted with two health personnel: a consultant
212 endocrinologist and the assistant director of nursing in the UCH diabetes clinic. In-depth

8

213 individual interviews were held with thirty diabetes mellitus patients in the outpatient ward of the
214 endocrinology clinic. The former lasted less than an hour for each participant, while the latter
215 lasted about an hour each. Table 1 reveals data on the socio-demographic characteristics of
216 the participants. While analyzing data, two key themes emerged: The perception of Diabetes
217 Patients and UCH health officials on the COVID-19 Pandemic and the coping mechanisms
218 employed by DM patients and the UCH clinic during the pandemic.
219 **Table 1: Demographic characteristics of participants (*n* = 32)**

| **Variable** | **Male Participants** | | **Female Participant** |  |
| --- | --- | --- | --- | --- |
|  | **(*n =9*)** | | **(*n =23*)** | |
| **Instrument** |  |  |  |  |
| KII |  |  | 2 |  |
| IDI | 9 | (30%) | 21 (70%) |  |
| Total (%) |  |  |  |  |
| Health personnel’s |  |  | 11 years (1) | |
| years of experience |  |  | 22 years (1) | |
| **Age** |  |  |  |  |
| 20-30 | 1 (11%) | | 2 (9.5%) |  |
| 31-40 | 0 | (0.0%) | 1 (4.7%) |  |
| 41-50 | 0 | (0.0%) | 5 (24%) |  |
| 51-60 | 0 | (0.0%) | 2 (9.5%) |  |
| 61-70 | 3 (33%) | | 3 (14%) |  |
| 71-80 | 5 (55%) | | 5 (24%) |  |
| 81 and above | 0 | (0.0%) | 1 (4.7%) |  |
| **Marital status** |  |  |  |  |
| Single | 1 (11%) | | 1 (4.7%) |  |
| Married | 8 | (89%) | 16 (76%) |  |
| Separated | 0 | (0.0%) | 1 (4.7%) |  |
| Widow | 0 | (0.0%) | 2 (9.5%) |  |
| **Religion** |  |  |  |  |
| Christianity | 6 | (67%) | 14 (67%) |  |
| Islam | 3 (33%) | | 6 (29%) |  |
| **Educational Status** |  |  |  |  |
| Tertiary | 5 (56%) | | 11 (52%) |  |
| Secondary | 2 (22%) | | 2 (9.5%) |  |
| Primary | 1 (11%) | | 2 (9.5%) |  |
| No formal Education | 0 | (0.0%) | 4 (19%) |  |
| **Occupation** |  |  |  |  |
| Professional/technical/managerial | 1 (11%) | | 6 (29%) |  |
| Clerical | 1 (11%) | | 1 (4.7%) |  |

9

| Sales and service | 1 (11%) | 9 (43%) |
| --- | --- | --- |
| Unemployed | 1 (11%) | 3 (14.2%) |
| Retired | 5 (55%) | 2 (9.52%) |
| **Socio-economic status** |  |  |
| High | 0 (0.0%) | 0 (0.0%) |
| Average | 2 (22%) | 6 (29%) |
| Low | 1 (11%) | 2 (9.52%) |
| **Year diagnosed with Diabetes** |  |  |
| 2-6 years ago | 5 (55%) | 6 (29%) |
| 7-11 years | 2 (22%) | 2 (9.52%) |
| 12-16 years | 1 (11%) | 3 (14.2%) |
| 17-21 years | 0 (0%) | 7 (33%) |
| 22-26 years | 1 (11%) | 1 (4.7%) |
| 27 years and above | 0 (0.0%) | 2 (9.52%) |
| **Year participant started attending UCH** |  |  |
| 2-6 years ago | 5 (55%) | 8 (38%) |
| 7-11 years | 1 (11%) | 3 (14.2%) |
| 12-16 years | 2 (22%) | 4 (19%) |
| 17-21 years | 0 (0.0%) | 2 (9.52%) |
| 22-26 years | 0 (0.0%) | 0 (0.0%) |
| 27 and above | 0 (0.0%) | 1 (4.7%) |
| **Diabetes type** |  |  |
| Type 1 | 1 (11%) | 1 (4.7%) |
| Type 2 | 7 (78%) | 6 (29%) |
| No knowledge about the type | 1 (11%) | 14 (67%) |

220

221

222 Table 2 presents the major themes and sub-themes that emerged from the study.
223 **Perception of diabetes patients and UCH health professionals on the COVID-19 Pandemic**
224 This theme contains three sub-themes (clinic appointments, effects of the COVID-19 pandemic
225 on diabetes patients, and adherence to COVID-19 protocols).
226 ***Clinic appointments***
227 Data from the key informat interviews revealed that the pandemic was perceived as a
228 period that has negatively affected appointments, clinic attendance, and the
229 management of diabetes in the clinic. The endocrinologist stated revealed that: *COVID-*
230 *19 has affected diabetes management in that many patients have had their appointments*

10

231 *rescheduled because we do not want so much contact between patients so that*
232 *asymptomatic patients will not infect other patients. Most of our patients now have*
233 *longer clinic appointments. Many of them do not get to talk to their health care provider,*
234 *that is, their doctors. They stop medications at will because nobody is really monitoring*
235 *them. For example, at the beginning of the lockdown, all appointments were cancelled,*
236 *and they had to be rescheduled for the beginning of this year, and we were seeing*
237 *patients that needed to be seen (KII, Consultant Endocrinologist).*

238

239 ***Effects of the COVID-19 pandemic on diabetes patients***
240 This sub-theme has ten codes which include the vulnerable groups during the pandemic. Some
241 of the participants revealed a clear understanding of the fact that diabetic patients are
242 vulnerable during the pandemic. Some opatients clearly knew the category of people
243 vulnerable to the COVID-19 virus. They perceived this category of people as the aged, those
244 who defy preventive measures, and people with underlying diseases. However, some
245 participants were unaware of who could be susceptible to the virus.
246 Some of the diabetic patients who were not observing the COVID-19 precautionary measures
247 stated that:
248 *Anybody can contract it, especially those who fail to wash their hands and follow the*
249 *preventive measures (IDI, Participant 2, Male, 75 years).*
250 *Those who fail to adhere to the necessary precautions, those who talk with their whole*
251 *mouth open without using their nose masks and those who stay too close to others (IDI,*
252 *Participant 9, Male, 21 years).*
253 *The elderly****/****people with underlying diseases Yes, people that are already sick before and*
254 *the elderly ones (IDI, Participant 10, female, 45 years).*
255 *It cannot have any negative impact on diabetes patients if they take their medications*
256 *regularly and follow necessary precautions (IDI, Participant 9, Male 21 years).*

11

257 Some participants, however, did not know who could contact the virus as evidenced in the
258 below excerpt:
259 *I can't say (IDI, Participant 1, female, 74 years).*
260 *Only God knows (IDI, Participant 4, female, 84years)*
261 They posited that the COVID-19 virus could have severe impacts on them. The opinion of one
262 participant in this regard is captured in the excerpt below:
263 *The diabetes patient could get infected and the person might not get cured of it. It could*
264 *even kill such a person (IDI, Participant 8, Female, 52years).*
265 A few others thought that COVID-19 cannot have any deadly implications on diabetes patients
266 since one can be treated if infected.
267 *The impact it can have is for one to get infected and even if one gets infected, since it can*
268 *be treated. I don’t think it can bring about any deadly impact except for someone whose*
269 *time is up on this planet earth already (IDI, Participant 12, Female 28years).*
270 Diabetic patients are not vulnerable in as much as they follow their diabetes regimen:
271 *I don't think there should be any effects if they use their drugs regularly, exercise as well*
272 *and take away the fear of contracting*
273 *the disease (IDI, Participant 2, Male 75 years).*
274 The in-depth interview further revealed that most of the participants were not afraid during
275 the pandemic. The submission of a participant is clear on this:
276 Undaunted include introductory sentence.
277 *Ehn…It did not make me scared because I firstly did not believe it was real but as time*
278 *went on and I started hearing that people were really contracting it, it was then I believed*
279 *that COVID-19 is real. However, till now, I have not seen anyone who has contracted it in*
280 *my vicinity. As such, that is one of the reasons I did not really get scared about it (IDI,*
281 *Participant 12, Female 28years).*

12

282 Some others were not fearful of the pandemic because they trusted in the Supreme Being.
283 The excerpts below capture this:
284 *I'm unruffled. I know we have problems all over the world but I'm a Christian and I'm*
285 *unruffled (IDI, Participant 2, Male 75 years).*
286 *I was not afraid, I don’t believe in it. I have Jesus (IDI, Participant 21, Male 65 years).*
287 Some others please reword. Redundance noted . Consider merging this statement with the introductory sentence on ‘ fearful of the pandemic …Supreme Being” were not afraid during the pandemic due to their trust in their medications, the
288 quality of health care provided in the UCH diabetes clinic and their proper adherence to the
289 COVID-19 precautionary measures. On the contrary, a few participants perceived the period
290 to be fearful. This is evident in the excerpts below:
291 A fearful period
292 *The only thing I can say about the impact here is the fact that when COVID-19 was*
293 *identified in Nigeria and there was a lock down, patients were not willing to come to clinic*
294 *(KII, Assistant director of nursing).*
295 *I was thinking I was going to die (IDI, Participant 14, Female 30years).*
296 *I was very scared but God's grace is sufficient (IDI, Participant 1, Male 74years).*

297

298 ***Adherence to COVID-19 Protocols***
299 This sub-theme was analyzed into two codes: physical presentation of patients only when
300 necessary and allowance of physical presentation of patients with strict adherence to COVID-
301 19 protocols.
302 Findings from the key informant interviews revealed that the physical presence of DM
303 patients during the pandemic is not a safe idea.Patients were advised to call doctors on the
304 clinic lines except when there is a pressing health concern that requires a physical
305 presentation of the patient in the clinic. Also, the opinion of the head nurse revealed that
306 patients are made to adhere to the COVID-19 precautionary measures whenever they need to
307 be physically present in the clinic. These opinions are encapsulated in the opinions below:

13

308 It should only be encouraged when it is necessary
309 *That is why we are reducing the numbers. If you look at our corridors, you don’t find*
310 *patients sitting on the corridor. If you came here two years ago, the whole corridor would*
311 *be filled with patients. People with poorly controlled diabetes are susceptible to a lot of*
312 *infections including COVID-19 because of poor glycemic control so what we do is to try*
313 *and optimize, control and reduce reasons why they have to keep moving up and down.*
314 *For example, coming to the clinic so, it is to their own advantage to call in our phone lines*
315 *and call when they have issues otherwise, there are patients that we really need to see*
316 *physically that we ask to come so the frequency of visit definitely has to be cut down to*
317 *avoid cross infection among diabetes patients (KII, Consultant Endocrinologist).*
318 It is not a bad idea if the *COVID-19* precautionary measures and protocols are duly observed.
319 *We as nurses have ensured that we make the environment good and safe for them to*
320 *come and of course we educate them. These are the protocols we have to observe when*
321 *we come to the clinic and most often than not, they comply like washing of hands, using*
322 *of sanitizer and wearing of nose masks and that is very paramount for them to do and*
323 *they have been complying. Most of them come with their mask on and they are ready to*
324 *comply. Also, the practice of social distancing is ensured. Before the pandemic about six*
325 *to seven persons can sit on a row but now, we ensure that only two patients sit on a row*
326 *and they have been complying(KII, Female Assistant director of nursing).*
327 **Coping mechanisms adopted by diabetes patients in**
328 ***Mechanisms adopted by the clinic***
329 Also, on the part of the diabetes clinic, mechanisms such as adopting an online database,
330 provision of avenues to review prescriptions, encouragement of daily monitoring of blood
331 sugar, telehealth, and awareness creation (Table 2).
332 Consider introductory sentences like: Coping mechanisms emerged as the third theme. The sub-themes were:..........The HCPs highlighted that....this narrative is supported the following quotes
333 Adoption of an online database include introductory sentences before direct quotes
334 *Now that they're using technology, things are improving so little by little I hope they will*
335 *do better (IDI, Participants 11, Female 65 years).*

14

335 Provision of avenues to review prescriptions
336 *Well, if people want their prescriptions renewed without seeing the doctors, they pass*
337 *the prescriptions and we renew them (KII, Consultant Endocrinologist).*
338 *The endocrine clinic pays close attention to their patients. They interact with them both*
339 *on phone and one on one basis and patients are encouraged to monitor their blood sugar*
340 *glucose daily. Majority of them have their exercise book wherein they record and this has*
341 *been a very good idea (KII, Assistant director of nursing).*
342 Telehealth refer to previous comment on introductory sentence
343 *We have phone lines that run on Wednesdays between 9am and 11 am. All patients with*
344 *diabetes or diseases relating to diabetes can call in and give their complaints relating to*
345 *glucose control and we can easily modify their medications and attend to them on phone.*
346 *Although, the frequency of seeing patients has reduced, in a way, the care has improved*
347 *for people that called online, that is, instead of coming to the clinic or waiting for their*
348 *clinic day, they can discuss their issues on the phone and the needed care can be given(KII,*
349 *Consultant Endocrinologist).*
350 *The main thing we did is to ensure that the patients are able to communicate with their*
351 *caregivers. Some of them have their doctor’s phone number whereby they get in touch*
352 *with them and discuss issues relating to their health with them. During the lockdown, it*
353 *was pasted outside in front of our doors. Sometimes when patients came* then and were
354 agitating to see their doctors, *we tell them to call that number and they did. Those who*
355 *came during the lockdown have the doctors’ phone numbers most especially. Some*
356 *patients tell us that they have called the doctor and him or her gave them an appointment*
357 *for a particular day and once the doctor comes and we try and verify and the doctor truly*
358 *acknowledges the fact that he or she was the one who gave them an appointment for*
359 *that day over the phone (KII, Assistant director of nursing).*
360 Informing patients about the availability of the telehealth service

15

361 *Yes. The notices are outside on the doors and the records place showing the lines to call*
362 *and the day to call endocrinologists. The nurses and service control (SERVICOM) officers*
363 *also tell them (KII, Consultant Endocrinologist).*
364 ***Personal coping mechanisms adopted by DM patients***
365 The patients utilized positive coping mechanisms such as adherence to precautionary
366 measures and reliance on the media for COVID-19 updates. This is captured in the responses
367 below: was this verbalized by all or some of the participants?
368 COVID-19 precautionary measures
369 *They make sure we use our nose mask, they do temperature check before you come in,*
370 *even at the gate they tell you to put your mask on (IDI, Participants 20, Male 75 years).*
371 *I simply follow the laid preventive measures and I keep myself clean in the house’ (IDI,*
372 *Participant 4, Female 84 years).*
373 *I was just doing what I was supposed to do and didn't do what i wasn't supposed to. For*
374 *example, I have been avoiding crowded places. I have not attended Jumat service since*
375 *the pandemic began rather, my family and I observe our prayers together at home (IDI,*
376 *Participant 3, Male 63 years).*
377 Reliance on the media for updates on COVID-19 include an introductory sentence
378 *I listened to health programs on the radio and I adhered to their health advices and I also*
379 *took precautions. It was the precautionary messages and COVID-19 jingles delivered by*
380 *newscasters on the radio I listened and adhered to (IDI, Participants 8, Female, 52 years).*
381 *I am only aware of the fact that I got a message from the clinic during the early period of*
382 *the pandemic that I should call some numbers in case an emergency issue arises*
383 *concerning my health (IDI, Participants 8, Female 52 years).*
384 Most of the participants averred that though they were aware of the telehealth service
385 provided by the clinic, they did not utilize it as a coping mechanism. This is vivid in the opinion
386 of some participants.

16

387 *During the COVID I received text messages inviting me to come for my check up. But I*
388 *didn't come ooo!. But they're trying (IDI Participants 11, Female 65 years).*
389 *I didn't because there was no reason for me to call them (IDI, Participants 3, Male 63*
390 *years).*
391 Only a few patients opined that they were not aware of the telehealth service provided by the
392 clinic. The provision of the telehealth service was helpful as it came as a timely intervention
393 for some patients who utilized it. This is evident in the response of one of the participants who
394 opined thus:
395 *Yes during the pandemic when we wanted to see the doctor, we were told to call him on*
396 *the phone, when I called the doctor I was told the drugs to buy(IDI, Participants 30,*
397 *Female 44 years).*

398

399 ***Reasons for low subscription to telehealth***
400 The findings of the study revealed that despite the provision of telehealth service as a coping
401 mechanism, there are however some limitations to its use among diabetes patients in UCH.
402 *If a patient is coming to us for the first time, virtual is not the best way to be treated. It is*
403 *after the patient has been seen physically for some time in the clinic that he or she can*
404 *then subscribe to telehealth services. It is not a substitute for physical clinic presentation*
405 *but it is an alternative way to sort out issues so that the patient does not need to come*
406 *in to see physically. For example, we cannot manage the case of a foot ulcer on the phone*
407 *as such, such a patient has to come to the clinic for us to see the extent and whether he*
408 *needs surgical ablation or not (KII, Consultant Endocrinologist).*
409 Another noticed limitation to the use of telehealth service is lack of a phone
410 *Some of them might not have a phone, some of them may also not see any reason why*
411 *they should call if they do not have issues (KII, Assistant director of nursing).*

412

17

413 ***Dangerous mechanisms employed by DM patients in the clinic***
414 The study, revealed the employment of negative mechanisms by DM patients in the
415 clinic. This is evident in the excerpts below:
416 Reducing drug dosage
417 *Many of the patients have reduced the doses of their drug so that it will last longer, some*
418 *of them have stopped checking their blood glucose because the strips are more expensive*
419 *this is owing to the fact that their usual source of money is not forthcoming so they check*
420 *less than before (KII, Consultant Endocrinologist).*
421 Changing of medications to cheaper brands
422 *Some patients would also change their drug to a cheaper brand if they find out that there*
423 *is another drug for diabetes and it may necessarily not be the best option for them (KII,*
424 *Consultant Endocrinologist).*
425 Reliance on religious institutions rather than the clinic for health instructions
426 *Some of the time, the religious houses intensify issues as some patients are told that they*
427 *have prayed for them and that they are healed as such, they are asked to throw their*
428 *medications away. Before they know, the patient’s condition deteriorates and there may*
429 *not be a remedy by the time they come back to the hospital. I am talking from my*
430 *experience. This may kind of affect their health and life style while trying to manage*
431 *themselves(KII, Assistant director of nursing).*
432 The use of herbal medicines
433 *Some use local concoctions and most times, it does not help (KII, Assistant director of*
434 *nursing).*
435 *I used some traditional herbs to also compliment my medications. I drank the juice from*
436 *boiled mango leaves and ginger. I also took my injections. I stayed indoors and maintain*
437 *social distancing the few times I go out. Most times even if I feel like going out, my parents*
438 *will not allow me go out because they know that I am more vulnerable to COVID-19 (IDI,*
439 *Participants 12, Female, 28 years).*

18

440 *I do a lot of steaming, herbal steaming, I cook dongoyaro leaves and the bark, we boil it,*
441 *and we drink on the first day and subsequent days we just steam. Everybody in my house*
442 *steams, we use menthol, we add menthol to it and we take a lot of supplements, vitamin*
443 *C and D, that’s all we’ve done so far (IDI, Participants 17, Male 77 years).*

444

445 **Table 2: The codes extracted for the perception and coping mechanisms**

| **Theme** | **Sub-theme** | |  | **Code** |
| --- | --- | --- | --- | --- |
|  | Clinical appointment | | | Effect of COVID-19 on diabetes patients' |
|  |  |  |  | appointment |
|  | Effect of | the COVID-19 | | ^✓^ Vulnerable groups during the pandemic of |
|  | pandemic |  |  | diabetes patients |
| Perception |  |  |  | ^✓^ Fear |
|  | Adherence to COVID-19 | | | ^✓^ Physical presentation of patients only when |
|  | protocols |  |  | necessary |
|  |  |  |  | ^✓^ Allowance of physical presentation of |
|  |  |  |  | patients with strict adherence to COVID-19 |
|  |  |  |  | protocols |
|  | Mechanisms adopted by | | | ^✓^ Adoption of an online database |
|  | the clinic |  |  | ^✓^ Provision of avenues to review prescriptions |
|  |  |  |  | ^✓^ Encouragement of daily monitoring of blood |
|  |  |  |  | sugar |
|  |  |  |  | ^✓^ Telehealth |
|  |  |  |  | ^✓^ Adherence to COVID-19 protocols |
|  | Personal |  | coping | ^✓^ Adherence to COVID-19 protocols |
|  | mechanisms adopted by | | | ^✓^ Media advice |
|  | DM patients | |  |  |
|  | Reasons | for | low | ^✓^ Severe diabetes |
|  | subscription | | to | ^✓^ Lack of telephone and need to utilize the |
| Coping | telehealth |  |  | mechanism |
|  |  |  |  | ^✓^ Lack of need to utilize it |
|  | Dangerous mechanisms | | | ^✓^ Wrong or irregular medications |
|  | adopted by DM patients | | | ^✓^ Reliance on religious institutions for health |
|  |  |  |  | instructions |
|  |  |  |  | ^✓^ Usage of traditional medicines |
|  | Suggested |  | coping | ^✓^ Health education and sensitization |
|  | mechanisms | |  | ^✓^ Provision of subsidized services |
|  |  |  |  | ^✓^ Reduction of waiting time |
|  |  |  |  | ^✓^ Continuation of diabetes association meeting |
|  |  |  |  | ^✓^ Provision of more call lines staff |
|  |  |  |  | 19 |

^✓^ Improvement of services provided by staff

^✓^ Improvement of facilities

446

447

448 ***Suggested coping mechanisms for the management of the UCH clinic and the government***
449 Some recommendations were given by the participants on how the management of the UCH
450 Diabetes Clinic and the government can assist in easing the stressful conditions of DM patients
451 during the COVID-19 pandemic. They are captured in the excerpts below:
452 Provision of health education, subsidization, and ensuring the availability of drugs
453 *We have been talking about this for years that all diabetes medications should be*
454 *subsidised as much as possible. In many countries, it is given for free. The other*
455 *alternative is for many of our patients to be on national health insurance. That would also*
456 *reduce the burden on the patients. The diabetes medications should be largely subsidized*
457 *because most of these medications are expensive and most of our diabetes patients are*
458 *also on drugs for other diseases such as hypertension and cholesterol and everything has*
459 *to be treated together. Therefore, government’s subsidization on the treatment of*
460 *diabetes and all the adjuncts are very important (KII, Consultant Endocrinologist).*
461 *I think they can educate the patients and make their treatment cheaper if not free.*
462 *However, we know the load on the government and the hospital as well. They can*
463 *encourage Non-Governmental Organizations to sponsor patients who cannot afford to*
464 *buy their drugs. The Diabetes Association of Nigeria too can be of help. The Diabetes*
465 *Association of Nigeria are trying because most of the time when we call them to do blood*
466 *sugar test for us, they do not collect money. I think this is part of what can be done to*
467 *make diabetes patients cope in this pandemic. Once a patient needs to get a medication*
468 *that is let’s say #5,000 and the patient has only #3,000, and they know that there is an*
469 *association that is willing to support them with the remaining amount, they will be willing*
470 *to come to the hospital (KII, Assistant director of nursing).*

20

471 *What I think can be done is….You see, there is a saying that someone who has diabetes*
472 *and does not want to die early needs money because it is not easy if I would be honest*
473 *with you. I think what they can do is if they can help us subsidize our drugs. If we are given*
474 *free drugs once in a while even if it is one drug, it would go a long way. What can be done*
475 *is if they can help us that way once in a while then if they can help us find means through*
476 *which our medications will not be scarce to get and they should help us such that it would*
477 *not be too expensive than what we can bear. See… if it is not too expensive, there is*
478 *nobody that does not want to use their medications and be healthy but the inability to*
479 *afford the drugs makes one not to be faithful with the medications. Those are the ways*
480 *I think they can help us(IDI, Participant 12, Female 28 years).*
481 *They should provide free drugs for us. Many at times, most of us do not come to the clinic*
482 *because we do not have money(IDI, Participant 9, Male 21 years).*
483 Reduction of the consultation fee include introductory sentence
484 *One thing they can do is to reduce the consultation fees. Sometimes after paying the*
485 *money, there won’t be any money left. And sometimes if we pay and the doctor is not*
486 *around, the hospital won’t refund the money, that one is gone(IDI, Participants 30,*
487 *Female 44 years).*
488 *We are not equal, and not all buoyant. The consultation fee can be reduced and some*
489 *things can be given for free, even if it is a few drugs (IDI, Participants 13, Female 49 years).*
490 Reduction of waiting time include introductory sentence
491 *Ah the major thing is that they should answer us in time. I understand that they will first*
492 *do ward round but immediately they come back they should answer us (IDI, Participants*
493 *11, Female 65 years).*
494 Provision for the continuation of the UCH diabetes association meeting include introductory sentence
495 *We have an association but UCH is not allowing us to hold meetings now, and its affecting*
496 *a lot, through the association, we have had numerous lectures from physiotherapist and*
497 *dieticians, to teach us more about our condition but our venue is small and due to COVID-*

21

498 *19 regulations, those meetings can’t hold but we have pleaded with the management to*
499 *allow us to do it outside in the open. Because the absence of those meetings is affecting*
500 *some of us who have no clue as to go about something regarding our ailment (IDI,*
501 *Participants 26, Female 68 years).*
502 Other suggested coping mechanisms include opening more call lines to ensure efficient
503 telehealth services, ensuring patients are treated politely in the clinic, and provision of
504 hygienic toilet facilities.

505

506 **Discussion Consider including countries where studies were published in this section. This will give a comprehensive picture to the reader without having to go to the reference list**
507 This study sought to explore the perception and coping mechanisms employed by DM
508 patients during the COVID-19 pandemic. We found that most participants were not fearful due
509 to the pandemic but were optimistic while they played their part in ensuring they were safe.
510 This is consistent with the findings of Sukanya and colleagues [18], which revealed that most
511 of the participants in their study were not so anxious about the pandemic but were rather
512 optimistic. Also, the participants in this study demonstrated a clear understanding of those
513 vulnerable to COVID-19, as some posited that anybody who defies the COVID-19 precautionary
514 measures, people with underlying diseases such as diabetes, are susceptible to the virus. This
515 attests to the supposition that COVID-19 sensitization and training were done in communities,
516 and health facilities and surveillance mechanisms in communities were improved in Nigeria
517 [19].
518 Literature has revealed that some health facilities had to shut down the entire clinic, including
519 diabetes outpatients, to protect the health care providers and patients from contracting the
520 virus [20]. Similarly, our findings show that the vulnerable nature of the patients informed
521 why all appointments in the UCH diabetes clinic were cancelled during the early period of the
522 pandemic, after which they were rescheduled till January 2021. Furthermore, our study
523 confirms compliance with WHO’s [4] recommendation on the use of telemedicine in other to
524 bridge the health access gap caused by the pandemic. Hence, the physical presentation of
525 patients during the pandemic was only encouraged when necessary. However, the study also

22

526 revealed that patients who attend the diabetes clinic have been adhering to the COVID-19
527 precautionary protocols such as wearing masks and social distancing most especially
528 when they attend the clinic since it reopened.
529 Contrary to the assertion made by Ahmed [5] that most of the public hospitals have been
530 converted to COVID-19 treatment centers, which made lots of patients with other diseases
531 stranded when they needed medical attention, this study found that the UCH diabetes clinic
532 was only closed during the early period of the pandemic in other to ensure the safety of its
533 staff and patients. In addition, the study revealed that the patients were not left stranded by
534 the clinic as telehealth was provided as an alternative Patients also had access to
535 healthcare personnel on an appointment basis after the clinic reopened in January 2021.
536 Interestingly, the study revealed that patients were encouraged by the clinic to monitor their
537 blood glucose daily by keeping records of it in a book. Another measure instituted was
538 reducing the crowd in the outpatient clinic by extending patients’ appointment dates. This
539 had the negative effect of limiting access to accessing healthcare personnel during the
540 pandemic. As a result, millions of diabetes patients have had their routine screening deferred
541 [11].
542 The findings from the in-depth interviews were consistent with those of the key informant
543 interviews. Both revealed that COVID-19 precautionary measures were strictly adhered to in
544 the clinic, and patients have been compliant. This is, therefore, consistent with the
545 recommendations of the WHO that preventive measures such as social distancing, the use of
546 masks, and hand sanitisers should be adhered to [4]. Awareness of the availability of the
547 telehealth service was high among the participants. However, awareness goes beyond
548 knowing that a service exists. It is concerned with understanding and utilizing that knowledge
549 [21].
550 Contrary to the perceptions of the key informants that some patients may not utilize the
551 telehealth service because they may not have a phone. Findings from the in-depth interviews
552 revealed that most patients did not utilize telehealth because they did not have reasons to
553 use it. This contradicts the findings of Hartmann-Boyce [22], which showed that many
554 patients could not utilize the telehealth service because of their inability to afford the

23

555 equipment needed for the process. However, very few participants who claimed to utilize the
556 service in this study reported that it was helpful.
557 The literature revealed that the limitations of telehealth service include the fact that it
558 prevents health practitioners from noticing helpful non-verbal communication of their
559 patients [23]. Another limitation recorded in the literature is that health practitioners often
560 omit certain assessments unconsciously and have incomplete assessments. The WHO only
561 recommended telehealth as a tool without specific training on utilising it efficiently. The
562 findings of this study have, however, extended literature as it showed that telehealth could
563 only be used for extant patients and not new patients as they have to present themselves
564 physically in the clinic while follow-up assessments can be done using telehealth. In addition,
565 the study found that another limitation of telehealth is that chronic complications of diabetes,
566 such as foot ulcers, cannot be managed using telehealth services.
567 The religious nature of Nigerian society is evident in the findings of this study, as most of the
568 participants found succor from the worries and fears of the pandemic in the Supreme Being.
569 This is consistent with the supposition that most diabetes patients eased their fear during the
570 pandemic by trusting the divine being and seeking supernatural protection [24]. Reliance on
571 the media for sensitization and updates on the pandemic was noted in the study. This affirms
572 the views of Effiong [25] that the media was utilized to pass messages to the masses on how
573 the virus can spread and how it can be prevented through regular washing of hands with soap
574 and water, social distancing, use of nose masks and sanitisers. Some patients coped by being
575 regular on their medications. This finding is consistent with similar studies [26, 27], as it also
576 revealed that some diabetes patients in UCH were able to cope when the clinic was closed
577 during the early period of the pandemic by using their last prescriptions before the pandemic
578 to procure more drugs.
579 Furthermore, the use of traditional herbs alongside medications was shown in the study. This
580 is consistent with the findings from the key informant interviews. This confirms that most non-
581 COVID-19 patients relied more on local medications and homemade remedies to cater for their
582 health during the pandemic [28]. Literature reveals that the burden of diabetes management
583 is more on DM patients in Nigeria, as about 74.5% of the health care expenditure is self-

24

584 financed by patients while the government provides only 25.5% [29]. This study found that
585 interventions such as subsidization, availability of drugs, and reduction of consultation fees
586 would help patients cope better during the pandemic. In addition, approval of the resumption
587 of the UCH diabetes association meeting, shortening waiting time in clinics, creation of more
588 call lines, polite treatment of patients, increased sensitization, and the provision of hygienic
589 toilet facilities would help alleviate the stress of the pandemic on diabetes patients in UCH.

590

591 **Strengths and Limitations**
592 The study's main strength is using key informant interviews and in-depth interviews to
593 examine the perception and coping mechanisms of DM patients during the COVID-19
594 pandemic. The qualitative nature of the study only permitted a small number of participants
595 in the hospital to be interviewed; therefore, it is important to evaluate our findings carefully
596 because extrapolating them to the entire country.

597

598 **Implications for public health research**
599 Based on the study's findings, it is imperative for health professionals to routinely conduct
600 psychological assessments for DM patients. Also, the health service managers must design
601 guidance and assistance for DM patients during and after the pandemic that considers factors
602 like risk perception, barriers, facilitators, and opportunities, to make it easier for them to
603 adopt coping mechanisms.

604

605 **Conclusion**
606 The study has shown that despite their condition, DM patients were not fearful of the COVID-
607 19 pandemic. DM patients were found to be adherent to the COVID-19 precautionary
608 protocols. The health systems' coping mechanisms to avert the pandemic's negative
609 implications were telehealth, encouragement of daily monitoring of sugar levels, and the
610 provision of avenues for a medication review. Additionally, the patients relied on mass media
611 advice and adherence to safety protocols to cope with COVID-19. Based on the study's

25

612 outcomes, the government and other healthcare stakeholders must reinforce the resilience
613 of DM patients by alleviating their health burdens during the pandemic. This could be done by
614 subsidizing the prices of drugs, tests, and consultation fees, improving the waiting and
615 appointment system in the clinic, creating an online presence for the UCH Diabetes
616 Association Office, and employing more health personnel in the clinic.
617 **Declarations**
618 **Ethics approval and consent to participate**
619 Ethical clearance was sought from the Ibadan/University College Hospital Ethics Committee
620 (UI/UCH EC) with approval number: UI/EC/21/0064. In this study, we complied with all the
621 ethical guidelines pertaining to using human participants and peculiar to qualitative studies.
622 We anonymized all the transcripts and audio files by giving them pseudonyms to remove any
623 personal information that may be used to identify the study participants. The participants in
624 the study were given an information sheet that included information on the objectives,
625 methods, potential risks and advantages, compensations, who to contact, and an affirmation
626 of confidentiality, privacy, and autonomy. The participants gave written consent by signing
627 the informed consent form, and no participant under 18 years of age was involved in this
628 study. This demonstrated that they had read and understood the terms of reference before
629 participating freely in our study. We also encrypted a passcode and locked the audio files and
630 transcripts to prevent unauthorised individuals from accessing the material.
631 **Consent for publication**
632 Not applicable.
633 **Availability of data and materials**
634 The dataset used and/or analysed is available from the corresponding author upon reasonable
635 request.
636 **Competing interests**
637 The authors declare that they have no competing interests.
638 **Funding**

26

639 This study received no external funding.
640 **Author contribution**
641 OOT developed the study concept. OOT, and WOB, conducted the interview and performed
642 the analysis; OOT, WOB, RGA, and OAB wrote the abstract, introduction, methods, and
643 discussion sections. All authors proofread the first draft of the manuscript, contributed
644 intellectually to the overall development, and approved the final version for submission.
645 **Acknowledgments**
646 The authors express their appreciation to the University College Hospital, Ibadan, for the
647 privilege of conducting the study within its diabetes clinic. We also thank everyone for their
648 various contributions and assistance during the study.
649 **Abbreviations**
650 COVID-19: Coronavirus Disease 2019; DM: Diabetes Mellitus; IDI: In-depth Interview; KII: Key
651 Informant Interview; NCD: Non-Communicable Diseases; UCH: University College Hospital;
652 WHO: World Health Organization
653 **Authors detail**

^1^Department of Sociology, University of Ibadan, Ibadan, Nigeria; ^2^Department of Public Health Medicine, School of Nursing and Public Health, University of KwaZulu-Natal, Durban, South

Africa; ^3^Department of Public Health, York St John University, London, United Kingdom; ^4^Department of Family and Community Health, Fred N. Binka School of Public Health, University of Health and Allied Sciences, Hohoe, Ghana; ^5^Endocrinology and Diabetes Division, Department of Internal Medicine, College of Medicine, University of Ibadan & University

College Hospital, Ibadan, Nigeria.

27

**References**

1. Organization WH. Global report on diabetes: World Health Organization; 2016.
2. Organization WH. Diabetes 2022 [3rd November, 2022]. Available from: [http://www.who.int/mediacentre/factsheets/fs312/en/.](http://www.who.int/mediacentre/factsheets/fs312/en/)
3. Programme UND. The Impact of the COVID-19 Pandemic in Nigeria: A Socio-Economic Analysis 2020 [4th November, 2022].
4. Organization WH. Rapid assessment of continuity of essential health services (EHS) during the COVID-19 pandemic 2020 [3rd November, 2022].
5. Ahmed W. Lockdown and diabetes–what actually happened. J Diab Metab Disord Control. 2020;7(4):116-7.
6. Usifoh SF, Ademola B. Assessment of Diabetic Patients in a Tertiary Hospital on Knowledge, Practice of and Attitude to Lifestyle Modifications. RADS Journal of Pharmacy and Pharmaceutical Sciences. 2020;8(1):7-13.
7. Uloko AE, Musa BM, Ramalan MA, Gezawa ID, Puepet FH, Uloko AT, et al. Prevalence and risk factors for diabetes mellitus in Nigeria: a systematic review and meta-analysis. Diabetes Therapy. 2018;9(3):1307-16.
8. Federal Ministry of Health. Strategies for Improving Diabetes Care in Nigeria (SIDCAIN) & World Diabetes Foundation. Diabetes and NCDs in Nigeria:Perspectives, Challenges & the Way Forward. 2017.
9. Devermont J. Wake-up Call: What Covid-19 Reveals about Elderly and NCD Care in Sub-Saharan Africa: Center for Strategic and International Studies (CSIS); 2020.
10. Awucha NE, Janefrances OC, Meshach AC, Henrietta JC, Daniel AI, Chidiebere NE. Impact of the COVID-19 pandemic on consumers’ access to essential medicines in Nigeria. The

American journal of tropical medicine and hygiene. 2020;103(4):1630.

11. Selvin E, Juraschek SP. Diabetes epidemiology in the COVID-19 pandemic. Diabetes care. 2020;43(8):1690-4.
12. Etukudoh NS, Ejinaka RO, Olowu F, Obeta M, Adebowale O, Udoudoh MP. Coronavirus (COVID-19); Review from a Nigerian perspective. Am J Biomed Sci & Res-9 (1). 2020.
13. Anyanwu MU, Festus IJ, Nwobi OC, Jaja C-JI, Oguttu JW. A perspective on Nigeria’s preparedness, response and challenges to mitigating the spread of COVID-19. Challenges. 2020;11(2):22.
14. Bolarinwa OA, Sakyi B, Ahinkorah BO, Ajayi KV, Seidu A-A, Hagan Jr JE, et al., editors. Spatial Patterns and Multilevel Analysis of Factors Associated with Antenatal Care Visits in Nigeria: Insight from the 2018 Nigeria Demographic Health Survey. Healthcare; 2021: MDPI.
15. Bolarinwa OA. Factors associated with access to condoms and sources of condoms during the COVID-19 pandemic in South Africa. Archives of Public Health. 2021;79(1):1-9.
16. Osahon I, Memudu A. Perception and Healthy Attitudes of Nigerians to Covid-19. Acta Scientific Medical Sciences. 2020;4(6):18-9.
17. (NCDC). NCfDCaP. NCDC COVID-19 report dashboard 2020 [cited 2020 July 1, 2020]. Available from: [https://covid19.ncdc.gov.ng/.](https://covid19.ncdc.gov.ng/)
18. Nachimuthu S, Vijayalakshmi R, Sudha M, Viswanathan V. Coping with diabetes during the COVID–19 lockdown in India: Results of an online pilot survey. Diabetes & Metabolic Syndrome: Clinical Research & Reviews. 2020;14(4):579-82.

28

19. Ohia C, Bakarey AS, Ahmad T. COVID-19 and Nigeria: putting the realities in context. International Journal of Infectious Diseases. 2020;95:279-81.
20. Azeez T. E. Endocrinology Practice in COVID-19 era: The Nigeria experience. . Journal of Clinical and Molecular Endocrinology 2020;5(2):18.
21. Saurman E. Improving access: modifying Penchansky and Thomas’s theory of access.

Journal of health services research & policy. 2016;21(1):36-9.

22. Hartmann-Boyce J, Morris E, Goyder C, Kinton J, Perring J, Nunan D, et al. Diabetes and COVID-19: risks, management, and learnings from other national disasters. Diabetes care. 2020;43(8):1695-703.
23. Kee KF, Schrock AR. Telephone interviewing as a qualitative methodology for researching cyberinfrastructure and virtual organizations. Second international handbook of internet research. 2020:351-65.
24. Ketema B, Kaba M, Addissie A, Kantelhardt EJ. Challenges and coping strategies in non-communicable disease management during COVID-19 in Addis Ababa, Ethiopia: a qualitative study. 2020.
25. Effiong AI, Nseobot IR, Johnny AE, Frank EI, Abere OJ, Essien MO, et al. Assessment of Nigerian television authority (NTA) ongoing programme awareness campaigns on corona virus in Nigeria. Electronic Research Journal of Social Sciences and Humanities. 2020;2.
26. Ghosh A, Arora B, Gupta R, Anoop S, Misra A. Effects of nationwide lockdown during COVID-19 epidemic on lifestyle and other medical issues of patients with type 2 diabetes in north India. Diabetes & Metabolic Syndrome: Clinical Research & Reviews. 2020;14(5):917-20.
27. Olickal JJ, Chinnakali P, Suryanarayana B, Ulaganeethi R, Kumar SS, Saya GK. Effect of COVID19 pandemic and national lockdown on persons with diabetes from rural areas availing care in a tertiary care center, southern India. Diabetes & Metabolic Syndrome: Clinical Research & Reviews. 2020;14(6):1967-72.
28. Ahmed SAS, Ajisola M, Azeem K, Bakibinga P, Chen Y-F, Choudhury NN, et al. Impact of the societal response to COVID-19 on access to healthcare for non-COVID-19 health issues in slum communities of Bangladesh, Kenya, Nigeria and Pakistan: results of pre-COVID and COVID-19 lockdown stakeholder engagements. BMJ global health. 2020;5(8):e003042.
29. Oyelami F, Ukoha CE, Olunuga O, Adelekan A. Attitudinal Disposition and Management Perception among Diabetes Mellitus Patients in Selected Hospitals in Ibadan, Oyo State, Nigeria. 2019.

29

Supporting Information

[Click here to access/download](https://www.editorialmanager.com/pone/download.aspx?id=33571266&guid=eb564002-664f-4051-92a6-91fae2339681&scheme=1)

[**Supporting Information**](https://www.editorialmanager.com/pone/download.aspx?id=33571266&guid=eb564002-664f-4051-92a6-91fae2339681&scheme=1)

[STROBE-checklist-v4-combined-PlosMedicine.docx](https://www.editorialmanager.com/pone/download.aspx?id=33571266&guid=eb564002-664f-4051-92a6-91fae2339681&scheme=1)

Supporting Information

[Click here to access/download](https://www.editorialmanager.com/pone/download.aspx?id=33571269&guid=6ee58b21-ed7d-41a6-9720-2f436e7eb616&scheme=1)

[**Supporting Information**](https://www.editorialmanager.com/pone/download.aspx?id=33571269&guid=6ee58b21-ed7d-41a6-9720-2f436e7eb616&scheme=1)

[MSC project apprroval letter.pdf](https://www.editorialmanager.com/pone/download.aspx?id=33571269&guid=6ee58b21-ed7d-41a6-9720-2f436e7eb616&scheme=1)
