# Supplementary material for: Perception and coping mechanisms of patients with diabetes mellitus during the COVID-19 pandemic in Ibadan, Nigeria
Source: PLoS One. 2024 Aug 27;19(8):e0309451. doi: 10.1371/journal.pone.0309451 (PMC11349176; doi:10.1371/journal.pone.0309451)
Supplement: Attachment — Submitted filename: Response to Reviewers Comments 10th January, 2024.docx [file pone.0309451.s004.docx]

**Response to Editor’s/Reviewers’ Comments**

**Editor’s comments**

1. Please ensure that your manuscript meets PLOS ONE's style requirements, including those for file naming. The PLOS ONE style templates can be found at
   <https://journals.plos.org/plosone/s/file?id=wjVg/PLOSOne_formatting_sample_main_body.pdf> and 
   <https://journals.plos.org/plosone/s/file?id=ba62/PLOSOne_formatting_sample_title_authors_affiliations.pdf>

Reply

This format is duly followed.

2. Please include a complete copy of PLOS’ questionnaire on inclusivity in global research in your revised manuscript. Our policy for research in this area aims to improve transparency in the reporting of research performed outside of researchers’ own country or community. The policy applies to researchers who have travelled to a different country to conduct research, research with Indigenous populations or their lands, and research on cultural artefacts. The questionnaire can also be requested at the journal’s discretion for any other submissions, even if these conditions are not met.  Please find more information on the policy and a link to download a blank copy of the questionnaire here: https://journals.plos.org/plosone/s/best-practices-in-research-reporting. Please upload a completed version of your questionnaire as Supporting Information when you resubmit your manuscript.

Reply

The questionnaire has been filled out and included as supporting information.

3. We note that you have indicated that data from this study are available upon request. PLOS only allows data to be available upon request if there are legal or ethical restrictions on sharing data publicly. For more information on unacceptable data access restrictions, please see http://journals.plos.org/plosone/s/data-availability#loc-unacceptable-data-access-restrictions.

In your revised cover letter, please address the following prompts:

a) If there are ethical or legal restrictions on sharing a de-identified data set, please explain them in detail (e.g., data contain potentially sensitive information, data are owned by a third-party organisation, etc.) and who has imposed them (e.g., an ethics committee). Please also provide contact information for a data access committee, ethics committee, or other institutional body to which data requests may be sent.

Reply

The data utilised in this research contain potentially identifying or sensitive patient information; data are owned by the Institute for Advanced Medical Research and Training (IAMRAT). Please get in touch with IAMRAT via cfalade@comui.edu.ng or the first author for data request and access.

b) If there are no restrictions, please upload the minimal anonymised data set necessary to replicate your study findings as either Supporting Information files or to a stable, public repository and provide us with the relevant URLs, DOIs, or accession numbers. For a list of acceptable repositories, please see http://journals.plos.org/plosone/s/data-availability#loc-recommended-repositories.

We will update your Data Availability statement on your behalf to reflect the information you provide.

Reply

This is a qualitative research design. More information regarding this has been provided above.

4. Please upload a Response to Reviewers letter which should include a point by point response to each of the points made by the Editor and / or Reviewers. (This should be uploaded as a 'Response to Reviewers' file type.) Please follow this link for more information: <http://blogs.PLOS.org/everyone/2011/05/10/how-to-submit-your-revised-manuscript/>

Reply

This has been done.

**REVIEWER 1:**
I reviewed an article entitled “Perception and coping mechanisms among diabetes mellitus patients at the University College Hospital, Ibadan, Nigeria, during the COVID-19 pandemic”. It was interesting to read. A few changes were required to improve the general structure of the work.

Response: Thank you.

The abstract is relatively long and may exceed the maximum number of words permitted. It might be enhanced by condensing the introductory and conclusion sections. The results should clearly state the themes and subthemes that emerged from the investigation in the first place and please provide a brief explanation for each subtheme.
Overall, you have justified your research nicely in the introduction. However, the method needs to be modified so that it answers the research's objectives and title.

Response: Thank you. We have made few revision to the abstract section. However, the abstract is within the range set by PLoS ONE.

The study's title clearly states that the research mostly featured Dm patients, and you are interested in learning about their perceptions and coping mechanisms while dealing with the management of DM and pandemic covid-19. However, you did include two professional healthcare workers as respondents in your method. Please explain why they were added.
How did you select participants for the qualitative study? Please give a citation for your response.
How did you assure the respondents' diversity?

Response: We have addressed these comments.

Please omit the word “only” in your description of study criteria. Restructure your statement to make the grammar more readable. Restructure your statement to make the grammar more readable.

Response: We have removed “only” from the study area.

Who developed the questions for data collection? How do the questions appear? Please give an example of the questions that were asked. Who verifies each of the questions before they are used? How many times did the verification procedure occur?

Response: The researchers (authors) developed the questionnaires, and the ethnographers assessed its ability to answer the objectives of the study. Pre-testing was done prior to the actual data collection by the researchers in a different facility not used for the data collection.

What if the appointment was cancelled or the participant was unable to keep to the schedule because the interview was held in a hospital setting? How many times did the interview take place in total? How did you conduct the interview? Did you use a local language or English? Please provide specifics.

Response: The interview spanned for at least more than a week, and the participants were informed about this exercise. Hence, the majority of the participants turned up for the interview.

In terms of data analysis, please describe in detail the thematic process that you used in the study. Who checked the interview verbatim and was it shown to the respondents before it was processed further?

Response: We have revised the analysis section.

The four requirements to grow trustworthiness and establish rigors were credibility (internal validity), transferability (external validity/generalizability), dependability (reliability), and confirmability (objectivity), how did you confirm all of these aspects?

Response: We have provided a section under the analysis section describing these four requirements.

Because the involvement of two healthcare personnel did not connect well with the title of the study, the study's results require significant restructure. You may incorporate their feedback into another research project. As a result, all of the themes and subthemes must be readjusted.

Response: The responses have been adjusted to include only in-depth interviews conducted with the DM patients.

Please offer specific demographic information about respondents using a qualitative method. This is necessary to see the variability of the individuals selected for the study.

Response: The authors have addressed this.

I will go over the discussion after I have the amended version of the work, especially if the results have been corrected.

Response: Thank you.

REVIEWER 2

This is an important study. The quality of the manuscript can be improved Several grammatical and spelling errors noted. Inconsistencies in the use of DM patients/ diabetes patients and sampling method noted. The recruitment, inclusion criteria for the health care professionals is not included. The findings section needs to be reworked. It is lengthy with repetition and no flow. Reporting the healthcare professionals and the patients' quotes under one theme without a clear description together with lack of introductory sentences before direct quotes distorted the findings section. Please revise. Please consider rewording the conclusion to make the statements shorter, clearer and more impactful to the reader. Kindly see an annotated copy with the comments and suggestions.

Response: We have revised the manuscript per the comments.
