# Supplementary material for: Perception and coping mechanisms of patients with diabetes mellitus during the COVID-19 pandemic in Ibadan, Nigeria
Source: PLoS One. 2024 Aug 27;19(8):e0309451. doi: 10.1371/journal.pone.0309451 (PMC11349176; doi:10.1371/journal.pone.0309451)
Supplement: Attachment — Submitted filename: Reviewers_replies_R2.docx [file pone.0309451.s005.docx]

**Reviewers' comments and replies**

**Reviewer #1:**

| **S/N** | **Comments** | **Replies** |
| --- | --- | --- |
| 1 | I am satisfied with the corrections made. The manuscript looks better and is easier to comprehend. Thank you so much for taking all the comments into consideration. Well done! | Many thanks for taking the time to review our manuscript. |

**Reviewer #2:**

| **S/N** | **Comments** | **Replies** |
| --- | --- | --- |
| 1 | Methods= study design: reference this information.  Data collection: It is not clear how the participants were recruited. please elaborate. Was there compensation associated with participation in the study? The roles of the researcher/s and research assistants are not clearly outlined. There 4 research assistants, so what were their roles? When was the interview guide pretested? On how many participants? What were the findings of the pretest? Were they included in the main study? | - In-text citations have been included in the study design. Page 5, lines 121 to 125. - More information has been added on how the participants were recruited, and interviewed and how much honorarium gift was given to each participant. Page 7, lines 162 to 169. - The role of the research assistants has been added. Page 7, lines 181 to 182. - Information about the pretest has been added on page 8, lines 193 to 196. |
| 2 | Data analysis: Repetition noted, please proofread and revise. Reference information on thematic data analysis. | All the repetition has been deleted and all sentences have been cited accordingly. Page 7/8, lines 183 to 192. |
| 3 | Rigour and trustworthiness: The information stated under rigour contradicts a statement made earlier that research assistants were trained anthropologists. Please ensure consistency. | The last information has been replaced with a suitable sentence. Page 9, lines 211 to 212. |
| 4 | Demographic data and characteristics: Line 237-239: Include the duration in years to avoid confusion and inconsistency. Repetition noted. | All the demographic data and characteristic sections have been rewritten. Page 9, lines 233 to 247. |
| 5 | Line 362, 392, 472 and 479: typo= please correct. | - Line 362 has been corrected now line 388 on page 15. - Line 392 has been corrected now line 419 on page 16. - Line 472 has been corrected now line 497 on page 20. - Line 479 has been corrected now line 507 to 508 on page 21. |
| 6 | Include an introductory sentence under telehealth. | An introductory sentence has been included. Page 16, line 398. |
| 7 | Table 2: write the themes in full | The themes have been written in full in table 2. |
